# Model-based estimation of QT intervals of mouse fetal electrocardiogram

**DOI:** 10.1186/s12938-022-01015-5

**Published:** 2022-06-29

**Authors:** Namareq Widatalla, Kiyoe Funamoto, Motoyoshi Kawataki, Chihiro Yoshida, Kenichi Funamoto, Masatoshi Saito, Yoshiyuki Kasahara, Ahsan Khandoker, Yoshitaka Kimura

**Affiliations:** 1 School of Medicine, Tohoku University, 2-1 Seiryo-machi, Aoba-ku, Sendai, Miyagi 980-8575 Japan; 2 Khalifa University, Abu Dhabi, UAE; 3 Kanagawa Children’s Medical Center, Yokohama, Japan

## Abstract

**Background:**

Abnormal prolongation in the QT interval or long QT syndrome (LQTS) is associated with several cardiac complications such as sudden infant death syndrome (SIDS). LQTS is believed to be linked to genetic mutations which can be understood by using animal models, such as mice models. Nevertheless, the research related to fetal QT interval in mice is still limited because of challenges associated with T wave measurements in fetal electrocardiogram (fECG). Reliable measurement of T waves is essential for estimating their end timings for QT interval assessment.

**Results:**

A mathematical model was used to estimate QT intervals. Estimated QT intervals were validated with Q-aortic closure (Q-Ac) intervals of Doppler ultrasound (DUS) and comparison between both showed good agreement with a correlation coefficient higher than 0.88 (*r* > 0.88, *P* < 0.05).

**Conclusion:**

Model-based estimation of QT intervals can help in better understanding of QT intervals in fetal mice.

## Introduction

The ventricular repolarization period of action potential (AP) is reflected as a T wave in the electrocardiogram (ECG). T wave is important for QT interval assessment which is considered a critical biomarker for cardiac abnormalities. QT interval is the interval starting from the QRS complex to the end of a T wave and it represents ventricular electrical systole [1]. In humans, abnormal prolongation in the QT interval or long QT interval syndrome (LQTS) is thought to be linked to Torsades de Pointes (TdP) [2] and sudden infant death syndrome (SIDS) [3]. LQTS can occur due to mutations in specific genes that make up the ionic channels responsible for the cardiac AP, and disruptions in the same channels may lead to an abnormal prolongation in the AP duration (APD) [4].

The relationships in LQTS and SIDS is not well established [5], therefore, more research is needed to understand how LQTS leads to SIDS. Since genetic manipulation of humans is not possible, animal models, such as mouse models, can be used for better understanding of cardiac abnormalities and LQTS. Up until now, fetal QT intervals in mice have received minimal attention because of the challenges associated with T wave measurements. T waves are known for their low amplitudes which make their detection from fetal ECG (fECG) challenging [6]. Furthermore, a mouse fetal heart is much smaller than a human fetal heart which imposes additional challenges on measuring T waves. Due to the difficulties in measuring T waves in fetal mice, model-based estimations of the end of T waves can help in better understanding of QT intervals in mice.

In this study, a mathematical model for the estimation of end of T waves of fECG collected from normal fetal mice is proposed. The model discussed in this study was developed from our earlier mathematical model that addressed fECG of humans [7]. The model was developed based on the similarity between the human’s repolarization phase of an AP and the discharging phase of capacitors in resistor–capacitor (RC) circuits. Similarly, the model in this study was developed due to the high similarities between mice AP and the charging and discharging phase of the capacitor [8]. Since there are differences in APD patterns between humans and mice, our earlier mathematical model was adjusted to accommodate for the difference.

In our previous study [7] model-based QT intervals had high agreement with QT intervals calculated from scalp fetal ECG (sfECG) and Q-aortic closing (Q-Ac) intervals calculated from pulsed Doppler records. In this study, we validated our model with Q-Ac.

## Results

RR interval values, measurers’ evaluation of Q-Ac intervals and their difference percentages, and model-based QT interval values and their difference percentages to Q-Ac values are shown in Table 1.

**Table 1:** RR interval, QT-Ac interval of measurer 1 and measurer 2, model-based QT interval and difference percentage calculations results

Subject	RR interval (ms)	M1^*^ Q-Ac interval (ms)	M2^**^Q-Ac interval (ms)	Difference (%) M1–M2	Model-based QT interval (ms)	Difference (%), QT–M1 Q-Ac intervals	Difference (%), QT–M2 Q-Ac intervals
Fetus 1 (*n* = 69)	264 ± 28	140 ± 12	140 ± 12	0.31	137 ± 8	2	1.7
Fetus 2 (*n* = 10)	256 ± 0.52	134 ± 5.4	134 ± 3.4	0.22	133 ± 1.9	0.6	0.82
Fetus 3 (*n* = 111)	273 ± 4	135 ± 5	133 ± 7.4	1.2	139 ± 1.5	4.5	3.2
Fetus 4 (*n* = 48)	276 ± 1.1	136 ± 6.8	136 ± 5.1	0.12	139 ± 1.2	2.3	2.4
Fetus 5 (*n* = 71)	390 ± 86	167 ± 16	168 ± 18	0.6	170 ± 20	1.7	2.3

Fetuses 1 and 2 belong to the same mother. Fetuses 3, 4 and 5 belong to the same mother

*ms* milliseconds, *n* number of beats

^*^Measurer 1

^**^Measurer 2

**Fig. 1:**
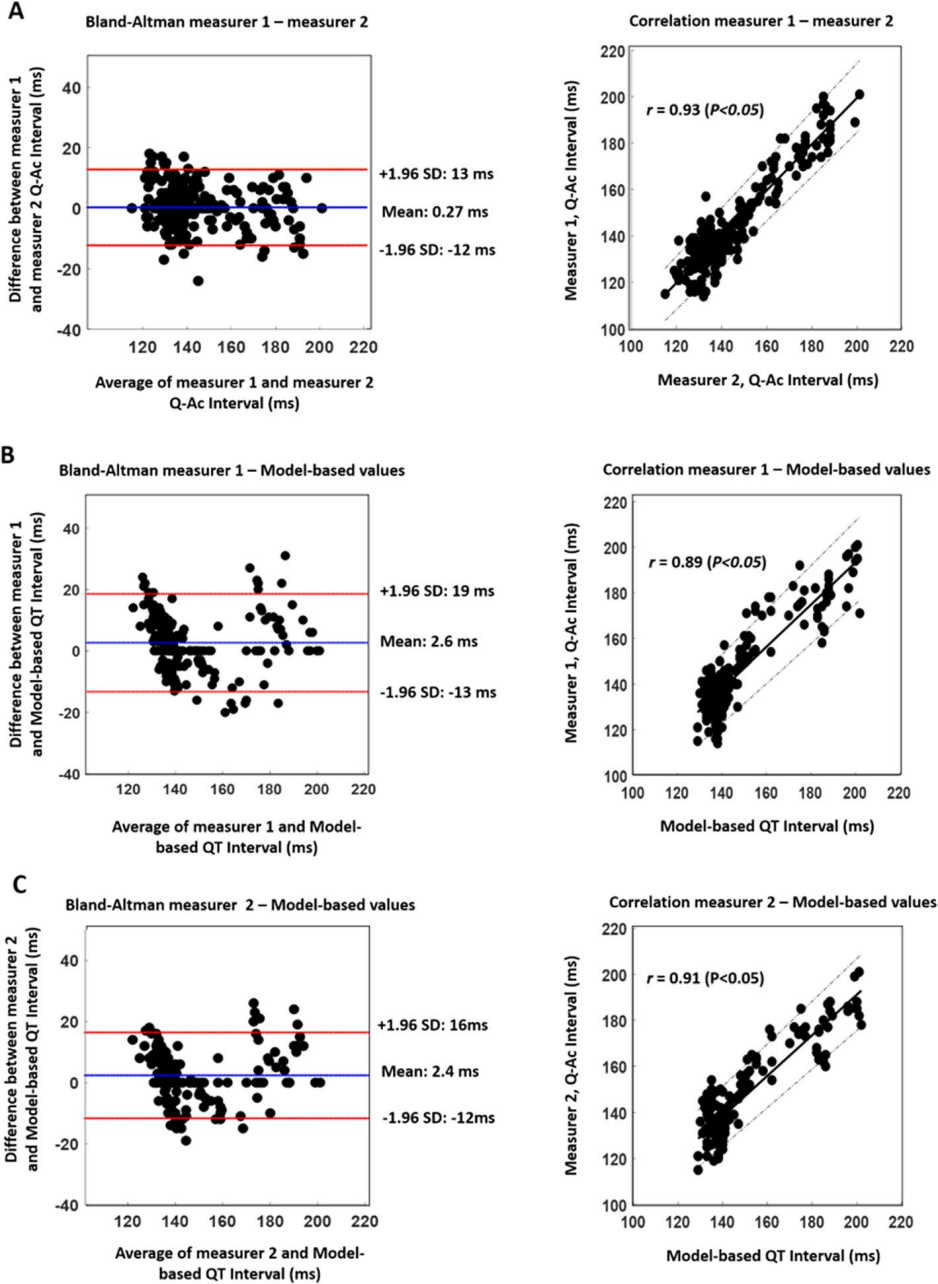
Bland–Altman and correlation analyses were performed to compare the Q-Ac interval values that were estimated by the two measurers **a**, and to compare model-based QT interval values with Q-Ac interval values of the two measurers **b**–**c**. QT and Q-Ac intervals were estimated beat by beat and the total number of heart beats was 309 from five fetal mice. **a** 95% of the values are within the limits of agreement (*r* = 0.93, *P* < 0.05). **b** 93% of the values are within the limits of agreement (*r* = 0.89, *P* < 0.05). **c** 92% of the values are within the limits of agreement (*r *= 0.91, *P* < 0.05)

In Table 1, the difference percentage for the measurers’ evaluation of Q-Ac is less than 5% which indicates high agreement. The difference percentage values between the two measurers’ Q-Ac values and the model-based QT intervals are also less than 5%. The low percentage difference between the measurers Q-Ac values and model-based QT intervals indicates that the model could estimate fetal QT intervals. For further validation of the measurers’ evaluation and model-based QT intervals, correlation and Bland–Altman (BA) analyses were conducted, and the results are shown in Fig. 1. In Fig. 1A, the correlation coefficient is high, *r* = *0.93* and the BA plot shows that at least 90% of the values are within the limits of agreement. High correlation coefficients and agreements are also shown in Fig. 1B, C which show the comparison between the measurer’s Q-Ac values and QT intervals.

## Discussion

The AP consists of depolarization, plateau phase and repolarization. APD patterns in murine ventricular myocardium differ from that of humans [9]. In mice, the APD decreases with fetal age, whereas the opposite occurs in humans [8]. In addition, mice APD is shorter than humans’ which explains the higher heart rates in mice [9]. Differences in the APD between the two species are mainly attributed to the differences in the ion channels responsible for the repolarization phase. The repolarization phase is controlled by the flow of potassium ion channels (K^+^) out of the cells [9, 10]. K^+^ currents are usually divided into transient outward currents (*I*_to_) and delayed rectifier currents (*I*_K_). *I*_to_ plays a major role in the repolarization phase in mice [11]; on the other hand, the same current is prominent in the plateau phase in humans. In adult mice, the *I*_K_ current, though still controversial, seems to be negligible. In contrast, the same current is important for the completion of the repolarization phase in humans [9]. According to L. Wang et al. [12], the *I*_k_ current is mostly prominent in fetal mice, and it reduces with fetal age and almost disappears during adulthood [12].

Due to the shorter APD in mice compared to humans, the model described in this study was adjusted to accommodate for the shorter repolarization period which signifies earlier occurrence of a T wave end. In our earlier model [7], the end of a T wave was calculated by subtracting a factor of $\frac{6\pi }{{x^{2} }}$ from the term $\frac{{{\text{mean}} \,\left( {R\left( t \right)} \right)}}{x}$ (Eq. 3). On the other hand, in mice, a factor of $\frac{2\pi }{{x^{2} }}$ was added to the same term to accommodate for the faster termination of a T wave in mice AP. To evaluate the accuracy of the model, the QT interval values were compared with the Q-Ac interval values estimated by two measurers from Doppler Ultrasound (DUS) records. The both measurers provided similar evaluations of Q-Ac interval with difference percentage less than 5% (Table 1). In addition, the similarity between the two measurers was further confirmed with correlation and BA plots in Fig. 1a which shows that around 95% of the values are within the limits of agreement with *r* = 0.91 (*P* < 0.05). After confirming the high agreement between the two measurers’ Q-Ac interval values, they were used for validating model-based QT-intervals. Table 1 reveals that the difference percentages between model-based QT intervals and Q-Ac intervals are less than 5%, and the correlation and BA analyses in Fig. 1b, c show high agreement between the same intervals with high correlation, *r* > 0.90 (*P* < 0.05). The mean values in the BA plots in Fig. 1b, c indicate that the model tends to provide QT interval values that are less than the Q-Ac interval values. The latter could be due the time difference between the mechanical activity and electrical activity of the heart [13]. Up until now, little information is available about the time lag between mechanical and electrical activities in fetal mice, therefore, it is difficult to further comment on the expected difference between the QT and Q-Ac intervals.

The model proposed in this study can be useful in thorough QT/corrected QT interval (QTc) studies which involve assessments of drugs that may cause LQTS [14]. For example, one can compare the predicted QT interval with the measured QT interval value after administration of a drug to determine if the drug is causing abnormalities to the QT interval or not. However, although the model showed high agreement with Q-Ac interval, it needs to be tested on more subjects. The model was tested on a low number of heart beats because of the difficulties that were involved in the experiment. Although the fECG recordings were carried out for 15 min, only heart beats that had clear pulsed Doppler images were selected for analysis in this study. Pulsed Doppler images of fetal heart were particularly challenging to obtain because of the small size of the fetal mouse. During the experiment, several attempts were made to obtain clear Doppler data and eventually only a few DUS images were clear enough for Q-Ac assessments.

## Conclusion

So far, it is unknown how repolarization patterns change in human fetuses. Therefore, it is important to utilize animal models, such as mice models, for better understanding of cardiac repolarization patterns. Repolarization patterns can be assessed by calculating QT intervals from ECG records. Nevertheless, due to the difficulties in measuring QT intervals in fetal mice, we proposed a model to estimate QT intervals by using RR intervals only. The model showed good accuracy with Q-Ac interval values of DUS. However, due to the low number of analyzed heart beats, more research is needed for further validation of the model.

## Materials and methods

The experimental protocol of this study was approved by the Center for Laboratory Animal Research, Tohoku University (animal experiment approval numbers are 2013 ido-501 and 2016 ido-079). The study protocol followed the Regulations for Animal Experiments and Related Activities of Tohoku University.

### Experimental procedures and data collection

C57BL/6N mice were purchased from CLEA Japan Inc. All mice had access to unlimited drinking water and were fed mouse pellets. All mice were kept in a room with a light–dark cycles of 12 h per cycle, in addition, mice were exposed to artificial light from 8:00 to 20:00. The room temperature was 22–26 °C, with a relative humidity of 50–60%.

2 female mice aged 7–20 weeks were housed together with 2 male mice of the same strain, separately, for one night; the next morning was considered a 0.5 embryonic day (E0.5). The experiment was performed at E18.5 and anesthetics were injected to maternal mice. The anesthetics composed of 0.2% ketamine and 0.05% xylazine for induction of anesthesia, and 0.5% isoflurane for maintenance of anesthesia. The maternal mice were kept on a supine position on a warm pad at 37 °C during the experiment. In addition, a far infrared heater was placed beside the maternal body to maintain a warm environment. Mice abdominal hair was removed using a hair removal cream (Veet, Reckitt Benckiser Group plc, Slough, England, UK). After confirming the mice were under anesthesia by disappearance of movements, the abdomen was open at the peritoneal cavity with fine scissors, and the uterus was exposed.

**Fig. 2:**
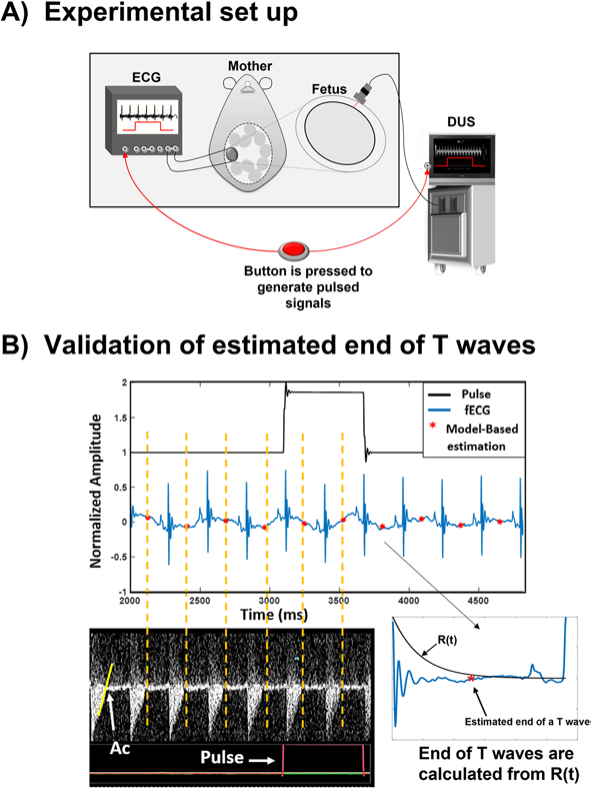
**A** fECG and pulsed Doppler images were obtained from the fetal mouse by using different systems. In order to match Doppler images with fECG records, a signal generator was connected to both systems to input pulsed signals. **B** The pulse signal that was recorded with fECG is aligned with the same signal that was recorded simultaneously with DUS. Q-Ac intervals were calculated by drawing vertical lines from the Ac timings of DUS to fECG. The red asterisks in fECG indicate the estimated end of T waves. End of T waves were calculated from *R*(*t*), Eq. 3. Ac timings were determined by drawing tangent lines where blood flow crosses the base line

The fECG was measured at a sampling rate of 1000 Hz for 15 min by attaching two electrodes, one at the fetal chest and the other at the back and fetuses were selected randomly (the ground was the maternal body). The fECG data were recorded by a portable multi-purpose bio signal amplifier monitoring system (Polymate AP1532 and AP Monitor; Miyuki Giken, Tokyo, Japan). Simultaneously, pulsed-wave Doppler measurement was conducted by using an ultrasound imaging system (VEVO 2100 Imaging System, FUJIFILM VisualSonics Inc., Tokyo, Japan). Since the fECG and DUS data were obtained using independent systems, it is necessary to match and align the two records afterwards for analysis. Hence, a pulse generator was connected to both systems to generate the same pulsed signals at random timings during the recording time (Fig. 2A). The timing at which the pulsed wave was generated, by pressing the switch button, was recorded in a sheet. fECG and DUS data were collected from a total of 5 fetuses that belonged to 2 mothers.

### Model description

The fetal mouse model is an adjustment to our previous fetal human model which is discussed in detail in [7]. Our previous model was developed based on the similarities between the capacitor discharging phase in a RC circuit and the repolarization phase of a human AP. Since the decay in repolarization phase of fetal mice AP looks similar to the discharging phase of the capacitor [8]., we made use of the equations that are used for the calculations of the discharging phase of the capacitor in a RC circuit, Eqs. 1,  2, to develop our end of T wave model:

1 $$ V \left( t \right) = v_{0} e^{{\frac{ - t}{{RC}}}} . $$

*t* is the time in seconds, *R* is the resistance in ohms and *C* is the capacitance in farads.

2 $$ RC = { }\frac{1}{{2\pi f_{c} }}. $$

*f*_*c*_ is the cut-off frequency in Hertz.

In Eq. 2, *f*_*c*_ was considered as similar to HR, hence the term $\frac{1}{fc}$ was replaced with RR since RR is the inverse of HR. Therefore, the term RC in Eq. 1 was replaced with the expression in Eq. 2 with replacing $\frac{1}{fc}$ with RR and setting *v*_*0*_ to 100 to obtain Eq. 3:

3 $$ R\left( t \right) = 100 e^{{\frac{ - 2\pi t}{{RR}}}} , $$

where *t* is the time in milliseconds and *RR* is the interval between the sequential R waves. By using Eq. 3, a constant was calculated to estimate an end of a T wave, which is denoted *k* as:

4 $$ k = \left| {\frac{{{\text{mean}}\left( {R\left( t \right)} \right)}}{x} + \frac{2\pi }{{x^{2} }}} \right|, $$

where *x* is the reciprocal of RR in seconds for one beat. The expression in Eq. 4 is slightly different than the one in our previous study to accommodate for the difference in HRs between mice and humans. In our previous mode, we subtracted a factor of $\frac{6\pi }{{x^{2} }}$ from the term $\frac{{{\text{mean}} \,\left( {R\left( t \right)} \right)}}{x}$. Finally, by using the constant *k*, the median of a range in which an end of a T wave, *T*_*E*_, is expected to exist was calculated by using Eq. 5:

5 $$ T_{E} = {\text{median}} \left( {k - 0.5 < R\left( t \right) < k + 1} \right). $$

### Model validation

QT interval values estimated by the model were validated by using DUS as was done in our previous study [7]. In DUS, the end of a T wave is believed to occur close to Ac [15], 16]. Hence, Q-Ac intervals were calculated and compared with QT intervals. For validation purposes, Q-Ac intervals were evaluated by two measurers. *R* and *Q* timings were determined automatically using a MATLAB code (based on “findpeaks” function) and *Q* timings were considered as the lowest peak preceding an *R* peak. Detected *R* peaks were checked manually to ensure that R peaks were detected properly. Ac timings were evaluated manually by drawing a tangent line at the point when the blood flow waveform crosses the baseline as indicated by the yellow line denoted as Ac in Fig. 2B. The Ac timing values were read from MATLAB by aligning the pulsed Doppler images with fECG tracings as shown in Fig. 2B. To read the timing values in MATLAB, a line (dotted yellow line in Fig. 2B) was drawn from Ac (Doppler) to the fECG signal (MATLAB) [lines were drawn by using Microsoft Power Point (Office 365)]. The exact values were ready by using the “Data tips” tool in MATLAB in which the values were displayed after clicking at a point in the fECG signal where the yellow dotted line matched with the Ac in Doppler. QT and Q-Ac intervals calculations were performed beat by beat and the total number of analyzed heart beats was 309 from five fetal mice. Before comparing Q-Ac intervals with QT intervals, Q-Ac intervals that were estimated by the two measurers were compared with each other to confirm consistency. The comparison was performed by calculating the difference percentage per fetus and performing BA analysis [17, 18] for all the 309 heart beats. Difference percentages were calculated by using Eq. 6:

6 $$ {\text{Difference percentage }}\left( {\text{\% }} \right) = \frac{{Q_{{\text{Ac - QT}}} }}{{Q_{{{\text{Ac}}}} }} \times 100. $$

Following the Q-Ac interval consistency analysis, Q-Ac intervals were compared with model-based QT intervals by using the same previously mentioned comparison analyses, difference percentage and BA plots.

## Data Availability

Data are available upon a proper request by contacting Dr. Yoshiyuki Kasahara: kasa@med.tohoku.ac.jp.

## Abbreviations

Ac: Aortic closing
APD: Action potential duration
BA: Bland–Altman
DUS: Doppler ultrasound
E0.5: Embryonic day 0.5
E18.5: Embryonic day 18.5
ECG: Electrocardiogram
fECG: Fetal electrocardiogram
*I*_to_: Transient outward currents
*I*_K_: Delayed rectifier currents
K^+^: Potassium ion channels
LQTS: Long QT syndrome
ms: Millisecond
RC: Resistor–capacitor
sfECG: Scalp fetal electrocardiogram
SIDS: Sudden infant death syndrome
TdP: Torsades de pointes
